# Contribution of Duplicated Nucleotide-Binding Leucine-Rich Repeat (NLR) Genes to Wheat Disease Resistance

**DOI:** 10.3390/plants12152794

**Published:** 2023-07-27

**Authors:** Yongchao Hao, Yinghua Pan, Wuying Chen, Muhammad Abdul Rehman Rashid, Mengyao Li, Naixiu Che, Xu Duan, Yan Zhao

**Affiliations:** 1 State Key Laboratory of Crop Biology, College of Agronomy, Shandong Agricultural University, Taian 271018, China; 2 Guangxi Key Laboratory of Rice Genetics and Breeding, Rice Research Institute, Guangxi Academy of Agricultural Sciences, Nanning 530007, China; 3 Department of Agricultural Sciences/Bioinformatics and Biotechnology, Government College University Faisalabad, Faisalabad 38000, Pakistan

**Keywords:** wheat, nucleotide-binding leucine-rich repeat, NLR genes, gene duplication, disease resistance

## Abstract

Wheat has a large and diverse repertoire of NLRs involved in disease resistance, with over 1500 NLRs detected in some studies. These NLR genes occur as singletons or clusters containing copies of NLRs from different phylogenetic clades. The number of NLRs and cluster size can differ drastically among ecotypes and cultivars. Primarily, duplication has led to the evolution and diversification of NLR genes. Among the various mechanisms, whole genome duplication (WGD) is the most intense and leading cause, contributing to the complex evolutionary history and abundant gene set of hexaploid wheat. Tandem duplication or recombination is another major mechanism of NLR gene expansion in wheat. The diversity and divergence of duplicate NLR genes are responsible for the broad-spectrum resistance of most plant species with limited R genes. Understanding the mechanisms underlying the rapid evolution and diversification of wheat NLR genes will help improve disease resistance in crops. The present review focuses on the diversity and divergence of duplicate NLR genes and their contribution to wheat disease resistance. Moreover, we provide an overview of disease resistance-associated gene duplication and the underlying strategies in wheat.

## 1. Introduction

Polyploid crops, such as wheat, triticale, oats, sweet potato, and peanuts, play a vital role in ensuring food security. Among these, the tetraploid durum wheat (pasta wheat, *Triticum durum*; 2n = 4x = 28) and the hexaploid bread wheat (*Triticum aestivum*; 2n = 6x = 42) are adaptable to various environmental conditions and, therefore, the most widely cultivated [1]. The production of wheat worldwide in 2021 was recorded to be as high as 781 million tons (FaoStat; https://www.fao.org/faostat/en/#data (accessed on 20 June 2023)). However, in spite of the high production, wheat and other crops are often threatened by abiotic and biotic stressors that significantly reduce their yield and quality. Specifically, diseases caused by pathogenic bacteria, viruses, fungi, and oomycetes have resulted in significant crop losses, affecting food security worldwide [2]. As in other plants, wheat resists pathogens via their multilayered innate immune responses: one mediated by pattern recognition receptors (PRRs) on the cell surface and the other mediated by nucleotide-binding leucine-rich repeats (NLRs) intracellularly [3,4]. Typically, plants sense pathogens via the immune receptors that detect the pathogen-derived molecules and initiate diverse defense responses interconnected to form a signaling network [5]. These PRRs, which are in the form of receptor-like proteins (RLPs) or receptor-like kinases (RLKs), detect conserved pathogen-associated molecular patterns (PAMPs) and activate pattern-triggered immunity (PTI) to instigate defensive responses against non-adapted pathogens. Additionally, the residual PTI provides basal resistance to the adapted pathogens [4]. Therefore, PTI is categorized as a non-race-specific resistance [4].

In order to overcome the impenetrable blockade of the first layer of the immune system, pathogenic organisms secrete a class of small molecules into plant cells called effectors [6]. Effectors of these pathogens subsequently interfere with PTI by hindering PRR translation, inhibiting the activity of PRRs and their complexes, affecting the transmission of MAPK and its downstream signals, and impacting vesicle transport and callose deposition [7,8,9]. In this way, pathogenic bacteria can successfully infect. However, plant cells will not surrender easily. Faced with this situation, plants will urgently activate the next immune layer. The NLR receptor within the cell can indirectly or directly spot the effector, causing the plant to initiate a second immune response, which is called effector-triggered immunity (ETI), and the effector recognized by the NLR protein is called avirulent (Avr) protein [10]. The ETI is usually accompanied by programmed cell death (PCD); this hypersensitive response (HR) is crucial for plant resistance to biotrophic pathogens [11]. In addition, the downstream immune response activated by ETI is similar to PTI but with greater intensity and longer duration [4]. Previous studies have found significant differences in recognition mechanisms and early signal transduction between PTI and ETI. Therefore, traditionally, people believe that PTI and ETI are independent systems [12,13]. However, recently researchers have found that multiple mutants of PRRs and their co-receptors in *Arabidopsis* have significantly lost plant ETI resistance triggered by multiple effector factors, suggesting that PRRs and their co-receptors are critical in activating NLRs [14]. In summary, PRRs and NLRs work together to enable plants to resist infection by most pathogens. However, pathogenic organisms will never surrender easily. They will continue to invent “new weapons” (effectors) to overcome the existing immune system and successfully infect plant cells. Similarly, plants will evolve new immune receptors to deal with the continuous and varied attacks of pathogenic bacteria [14,15,16,17].

## 2. NLR Gene Structure and Categories

**Figure 1:**
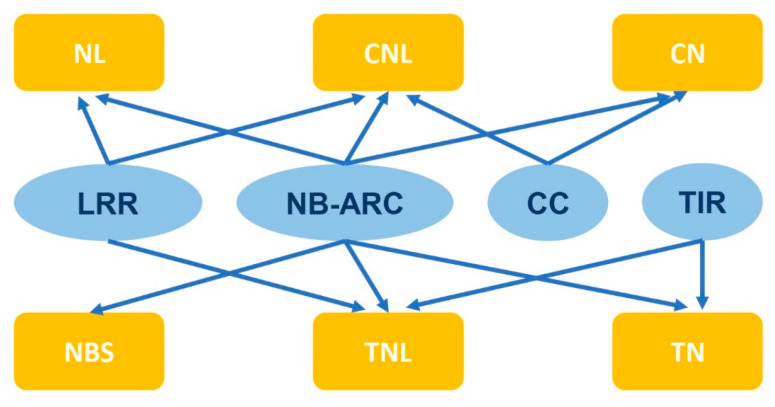
Subgroups of NLR genes based on domain structures.

Typically, the structure of NLR consists of three parts, including a variable N-terminal domain, a conserved, central nucleotide-binding adapter (NB-ARC) domain, and a C-terminal leucine-rich repeat (LRR) domain [18,19,20]. The variable N-terminal domain is divided into two categories based on the protein sequence and similarity to known proteins: Toll interleukin-1 receiver (TIR) and coiled coil (CC) [21,22]. The NLR genes are classified into subgroups depending on their domain architecture, including NBS, TN, TNL, CN, NL, and CNL (Figure 1) [23]. Some studies have indicated that NLRs with a deletion of the domain at the N-terminus cannot elicit plant immunity, while their expression alone is sufficient to induce HR responses [24,25]. Therefore, this domain of NLR genes is believed to stimulate and transmit immune signals downstream. In animal NLRs, when the effector is recognized, the entire NLR protein can oligomerize and form high molecular weight complexes known as inflammasomes to stimulate immune signaling [26]. In plants, several studies have reported that NLRs self-associate upon specific recognition of their corresponding effectors, including TIR-NLRs (tobacco N and *Arabidopsis RPP1*) and a CC-NLR (*Arabidopsis ZAR1*) [27,28,29]. The N-terminal TIR and CC domains may be crucial for NLR oligomerization and activation [30,31].

NB-ARC, the relatively large domain of NLRs, includes three subdomains: the NB, the ARC1, and the ARC2 subdomains. Among these, NB represents nucleotide binding, and ARC is so named because this domain appears in Apaf-1, R, and CED-4 proteins [32,33]. The NB-ARC domain is considered an ATPase domain due to its ability to bind with, exchange, or hydrolyze ADP or ATP nucleotides [34]. Generally, the NLR protein combined with ADP is in a closed or inactive state, and the combination with ATP promotes a conformational change in the NLR protein, turning it into an open or activated state. After NLR activation, it will transmit signals downstream to activate the plant’s immune system. Effector recognition by NLRs (directly or indirectly) will prompt NB-ARC to release ADP and bind ATP [14]. Additionally, this binding feature acts as a “molecular switch” model of NLRs in plant immunity [35]. The properties of the NB-ARC domain and its binding with different nucleotides form the structural basis of this “molecular switch” model [36]. LRR is named for the presence of multiple tandem leucine-rich repeats in this domain, and LRR domains regulate the activity of NLRs through intramolecular or intermolecular interactions [19,37]. Research has demonstrated that LRR domains negatively influence the activity of NLRs [38,39], and the LRR domain physically associates with the NB-ARC domain [40,41,42]. In addition, deletion of the LRR domain results in auto-activation [42,43]. However, other studies have shown that LRRs can also positively regulate the activity of NLRs, and self-activating mutations in potato Rx led to HR responses without pathogenic bacteria, whereas deletion of its LRRs suppressed HR responses [40].

## 3. Gene Duplication in Wheat

**Figure 2:**
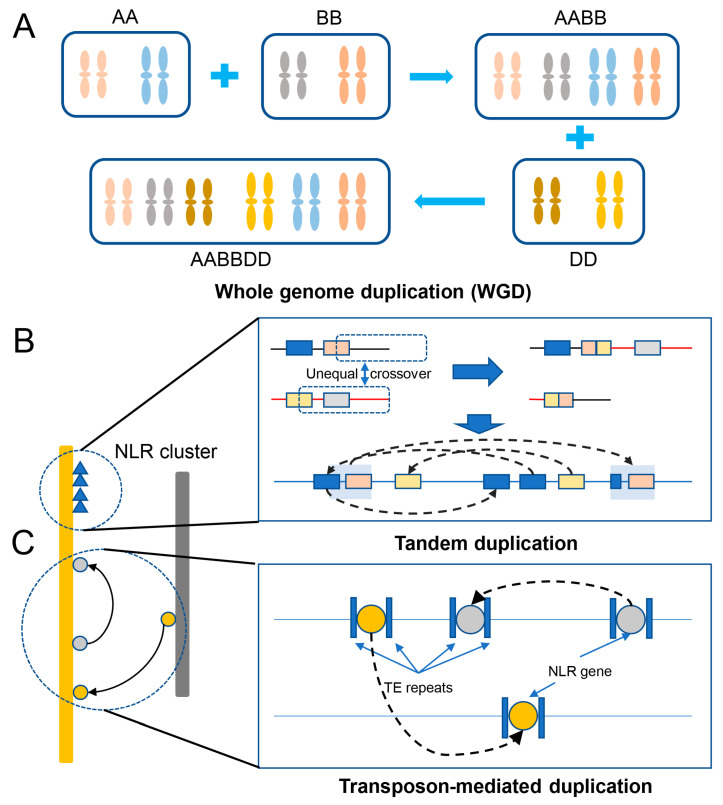
NLR duplication in wheat. (**A**) Whole genome duplication (WGD) through an increase in ploidy. (**B**) Tandem duplication through unequal recombination between similar alleles to form a gene cluster. (**C**) Transposon-mediated duplication of a gene associated with transposable elements (TE repeats) via replicative transposition to form dispersed duplicates.

The genome of bread wheat is one of the largest and most complex of all cultivated plant species. This complexity originated as a result of two rounds of historical whole genome duplication events [44] and recent small-scale duplications [45]. Gene duplication provides “raw genetic material” for the evolution of genes and gene functions to improve crops; it is also a key factor driving the evolution, domestication, and diversification of species [46]. Scientists have deciphered numerous large, complex, and highly repetitive genomes of various species of the *Triticeae* tribe [1,47,48,49,50,51,52]. Comparing the available genome sequences and functional genomic data of plants, we have gained immense knowledge of how genes are duplicated, how these duplicated genes gain novel roles, and their ultimate impact on genome evolution [53,54]. Among the various events or mechanisms, the duplication of the whole genome or an entire chromosome is the most extensive form of gene duplication [54]. Whole genome duplication (WGD or polyploidization) is an extreme mechanism that has benefited wheat’s complex evolutionary history; evolution of hexploid wheat through two hybridization and polyploidization events formed a new species with a huge genome and abundant gene set [55,56]. Then, hexaploid wheat spread worldwide (Figure 2A). About 55% of the bread wheat homologous genes exhibit 1:1:1 correspondence across the three homologous subgenomes, and another 15% possess a minimum of one gene copy in at least one of the subgenomes [8]. Tandem duplication or recombination is the event when two or more genes after duplication are positioned adjacent to each other on the same chromosome [54,57,58]. It is likely a major mechanism of NLR gene expansion to form NLR gene clusters in wheat (Figure 2B). Additionally, researchers found a recent burst of gene duplications in all sequenced species of the *Triticeae* tribe, and detailed analysis of the features of the gene duplication and their flanking sequences suggested the role of transposable elements (TEs) in causing this recent event [45]. In contrast to local tandem duplication, replicative transposition by TEs forms dispersed duplicates [59] (Figure 2C).

## 4. Diversity and Divergence of NLR Genes for Disease Resistance

Earlier research suggested great variation in the number of NLRs among closely associated species [60], and the number is not related to the size of the genome or the level of ploidy [61]. However, more than 1500 NLRs have been detected by analyzing the transcriptional and physical organization of the intracellular immune receptor repertoire in bread wheat [62]. More than 2000 NLRs have been identified using the fully annotated reference genome of bread wheat [50]. A pan-genome provides an opportunity to further study the numerical variation of wheat NLRs, and researchers used multigenome comparisons (11 wheat accessions) for characterizing NLRs and identified around 2500 loci with NLR signatures in each accession, while only 31–34% of the NLR signatures are shared across all genomes. They identified 5905 (98% identity) to 7780 (100% identity) unique NLRs in all wheat genomes, emphasizing the complexity and size of the immune receptor repertoire guiding disease resistance [1]. This may also demonstrate the relationship between NLR number and genome size and between NLR number and ploidy level. A recent study used a panel of 907 winter wheat accessions to construct a resistance gene atlas, and the majority of NLRs (96%) were grouped into 39,073 orthogroups, with the remaining being unique. Most orthogroups contained NLRs from at least 20 different accessions, but very few contained members from more than 500 accessions, which showed a different pattern of NLR diversity compared to the model plant *A. thaliana* [63].

Due to the diversity and abundance of pathogens in different environments, plants face dynamic selection pressure from habitat pathogens during their evolution, which results in the inability to maintain stable distribution of NLR genes between and even within species [12]. It has been found in research on different angiosperms that significant pathogen infection pressure has driven the expansion of NLR genes [64,65,66]. In the study of wild emmer wheat, it was found that changes in the NLR gene appeared to be rapid within the species [67]. Due to differences in the abundance of pathogenic bacteria in habitats, the population of wild wheat differentiated, with wild wheat growing in areas where powdery mildew was prevalent, then evolving resistance to powdery mildew [41]. As stated earlier, the clusters (medium and large) may vary greatly in their size among the ecotypes and cultivated varieties, indicating potential local adaptability [68,69]. For clusters containing highly homologous NLRs from an ancestral gene with few inversions, direct duplication of genes probably resulted in the original clustering; subsequently, increased rates of unequal crossing-over (UCO) during meiosis probably provided the material for rapid evolution and increased diversity in immune sensors, thereby expanding the cluster [70]. Under different pressures, these clusters rapidly contract or expand, which explains the large variations in cluster patterns between ecotypes [1,71]. However, the abundance of NLR genes is not solely beneficial; the more NLR genes plants maintain, the more health costs they incur [72]. Under no or less pathogen selection pressure, various plant species have exhibited contraction of NLR genes [73,74], leading to significant variations in the number and diversity of NLR genes between or within species.

Gene recombination is crucial for NLR diversification. Current evidence shows that the unconventional recombination (illegitimate recombination) between NLR genes is the main way to change the number of repeats of the LRR domain, which can cause a rapid increase or decrease in the repeat number of the LRR domain, further increasing the potential of NLRs to recognize different effectors [49]. In addition, if the NLR gene recombines with other genes (such as WRKY, NAC), it can cause the fusion of the domains of other genes to NLRs, further enriching the diversity of NLR structure and function (Figure 2B). For example, a WRKY domain is attached to the *Arabidopsis* NLR gene RRS1 at the C-terminus [75,76]. Further research found that this gene fusion phenomenon is very common in plant NLR genes [77]. In addition, some gene recombination events can also cause the loss of the NLR domain, resulting in truncated NLRs. Truncated NLRs are also ubiquitous in various plants and also play very important roles in plant immunity [25,78]. Interestingly, although the domains of truncated NLRs are incomplete, they can also perform similar functions to intact NLRs: they can directly or indirectly recognize effectors and induce plant immunity [45,78]. Taken together, gene duplication of NLRs provided diversity to cope with the infection of thousands of evolving pathogenic bacteria.

## 5. Duplicated NLR Genes Are Important for Wheat Disease Resistance

The duplication of NLR genes has several evolutionary implications. First, duplication can initiate evolution of new functions of the existing genes by facilitating accumulation of mutations. This change can lead to the evolution of new features and specificities of resistance, allowing plants to respond to a wider range of pathogen types [79]. Second, duplication can result in redundancy, where multiple copies of the same gene perform the same function. This redundancy can provide a buffer against loss-of-function mutations, ensuring the maintenance of a functional immune response [80]. Third, duplication can lead to subfunctionalization, where the duplicated genes acquire distinct functions through the partitioning of the original gene’s functions [81]. This process can result in the evolution of more complex immune responses, allowing plants to respond to a wider range of pathogen types.

Many studies have proven that the duplicated NLR genes contributed to wheat disease resistance. A new study has found that only one amino acid change can alter the specificity of the multiallelic wheat stem rust resistance locus *SR9*. The seven previously reported resistance alleles (*Sr9a*, *Sr9b*, *Sr9d*, *Sr9e*, *Sr9f*, *Sr9g*, and *Sr9h*) at the locus were characterized using a synergistic strategy. Among them, the *SR9H* protein is effective for the stem rust pathogen TTKSK (*Ug99*) and is only one amino acid different from *SR9B*. In addition, the resistance proteins of *SR9B* and *SR9G* also differ by one amino acid [82]. Researchers have isolated three major genes associated with yellow rust resistance in bread wheat (*Yr5*, *Yr7*, and *YrSP*), each with distinct recognition specificity. These resistance genes belong to an R gene cluster on the wheat chromosome 2B encoding NLR proteins [83]. Previously, the *Lr42* leaf rust R gene was transferred into bread wheat from the wild relative *Ae. tauschii*, where the *lr42* allele, four homologs of it, and four partial fragments of the gene were grouped as a cluster within an 871 kb region. We speculate that *Lr42* could have originated from an early locus, which expanded or maintained the copy number in other wheat species and barley. Typically, *Lr42* has an NLR structure and the “MAEAVVGQLVVTLGEALAKEA” homologous domain, similar to the MADA “MA(D/E)AxVSFxVxKLxxLLxxEx” motif of the known NLRs associated with rust (stripe, leaf, and stem rust) resistance in wheat [84]. The wheat progenitor *Ae. tauschii* has the NLR *YrAS2388* associated with Pst resistance on the 4DS chromosome. *YrAS2388R*, the Pst-resistant allele, has duplicate 3′-untranslated regions [85]. In synthetic hexaploid wheat, a mutation in the *YrAS2388R* allele disrupted Pst resistance, while the *YrAS2388R*-overexpressing transgenics were resistant to eleven races in common wheat and one in barley [85]. Additionally, the *Lr21* leaf rust resistance gene was introgressed from *Ae. tauschii* using a synthetic wheat species. Detailed analysis of the nucleotide sequence showed that the functional *Lr21* allele is a chimera of H1 and H2, two non-functional haplotypes of *lr21* [86,87]. On the other hand, bread wheat has only inactive *lr21* alleles; nevertheless, an active R gene could be reconstructed in vitro by intermolecular recombination of the two haplotypes.

Head-to-head or bidirectional gene pairs are common in eukaryotes and often include genes that are transcribed at similar rates; this specific property indicates the presence of common regulatory areas in the gene pairs [88]. Although there are few reports on wheat, research on other species can provide insights. In *A. thaliana*, the head-to-head cluster SOC3-CHS1-TN2 NLR has been observed to have co-expression and gene product interaction, and microarray data for other bidirectional gene pairs of NLR in *A. thaliana* support the proposal of shared regulatory regions [89]. Further experimental evidence is needed to establish this as a common trend for bidirectionally arranged NLRs; however, this justifies the genomic pattern.

## 6. Mechanism of NLR Gene Duplication for Disease Resistance

Meta-analysis of 314 cloned resistance (R) genes revealed that most genes encode intracellular or cell surface receptors [90]. These R genes encode proteins to induce or increase disease resistance (R) via nine distinct mechanisms. The mechanisms involve either direct or indirect recognition of pathogenic molecules on the cell surface with receptor-like proteins, leucine-rich repeat receptors, nucleotide binding, or via integrated domains; they also perceive transcription activator-like effectors (TALEs) by activating the executor genes or lose susceptibility through passive or active mechanisms or host reprogramming [90]. Researchers have elucidated the molecular mechanisms underlying the functions of a few R genes. However, elucidating the mechanisms in detail is necessary for engineering and adopting novel R genes [90]. The recognition of pathogen-derived *Avr* proteins by NLR proteins is a critical component of gene-for-gene or active defense, mostly occurring through the leucine-rich repeat (LRR) domain of the NLR proteins. In this process, the LRR domain specifically recognizes pathogen effectors, allowing the plant to initiate an effective defense response against the attacking pathogens. However, an interruption in disease resistance may occur due to mutations in *Avr* proteins, which consequently allows the pathogens to escape R protein-mediated molecular recognition [91]. This evasion mechanism enables pathogens to overcome the defense response of the plant, resulting in infection and disease incidence and further spread. Understanding the basis of this phenomenon is essential for designing durable resistance strategies in crop plants.

**Figure 3:**
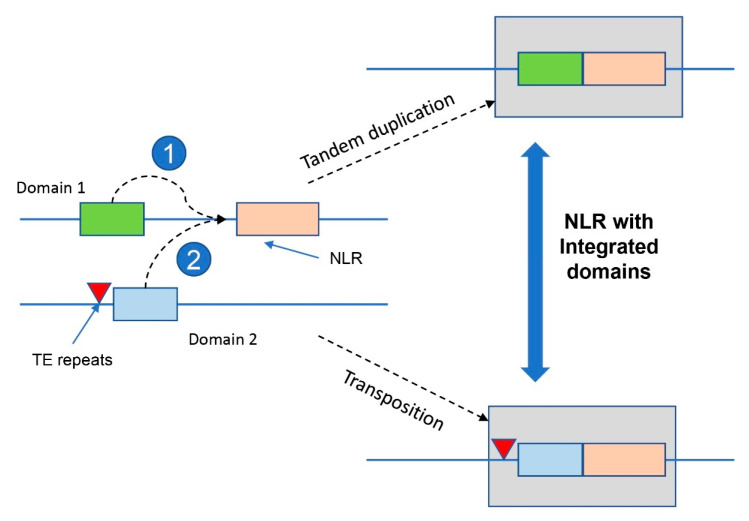
Origination models of NLR gene with integrated domains. (1) NLR gene with integrated domains generated by tandem duplication. (2) NLR gene with integrated domains generated by transposition.

Previously reported NLR resistance proteins have various additional domains, called integrated domains (IDs), that may confer novel functions or modulate NLR activity [92]. IDs can be derived from other immune-related proteins, such as kinases or transcription factors, and regulate the activation of receptors and signaling of the downstream components. Understanding the origin, evolution, and function of NLRs and IDs is crucial for elucidating plant immunity’s molecular mechanisms and developing novel crop improvement strategies [93,94,95]. For example, the *Arabidopsis* resistance protein *RRS1* has a WRKY domain, and the rice blast resistance protein *RGA5* has a RATX1 domain, both at the C-terminus; the wheat *Yr7*, *Yr5*, and *YrSP* stripe rust resistance proteins have BED domains at the N-terminus. In addition, the *YrU1* protein contains the ANK domain at the N-terminus and WRKY domain at the C-terminus [83,96,97]. We propose a hypothesis that genes with additional domains are likely to originate from duplication events in the genome, such as tandem duplication and transposon-mediated duplication; this will improve our understanding of the evolution of the NLR genes with integrated domains (Figure 3). NLR proteins can function together to trigger plant immunity and are classified into two groups: NLR pairs and sensor/helper NLRs [98]. NLR pairs are encoded next to each other in the same orientation and share a promoter. Paired NLRs tend to form complexes needed for immunity. Typically, one NLR has a decoy domain and acts as the sensor, interacting with pathogens. The other is a canonical NLR that transduces signals to activate immunity. NLRs with decoy domains are common in flowering plants, likely formed through ectopic recombination and transposition [13]. The other way NLRs interact is as a sensor or a helper. Among these NLRs, the helper presumably acts downstream of the sensor in immunity. Unlike the typical adjacent NLR pairs, the helper–sensor NLRs do not physically interact. In fact, they are usually not encoded at the same locus. However, the mechanism via which the sensors transmit signals to the helpers remains elusive [98].

A recent study reported that Rps11 in soybean provides broad-spectrum resistance to the oomycete pathogen *Phytophthora sojae*. In the genome, the giant NLR gene is positioned in a region carrying a large NLR gene cluster resulting from numerous unequal recombinations [99]. In wheat, several studies also reported that NLR clusters contributed to disease resistance. In the hexaploid wheat germplasm, *Pm3a–j*, a large multiallelic series of ten resistance alleles, each with distinct race specificity, accounted for the *Pm3* powdery mildew resistance [95]. Scientists have identified *Pm3* alleles in cultivars worldwide. After cloning *Pm3b*, the first allele [100], the remaining alleles were isolated by PCR and identified as a single gene’s true alleles in a cluster of *Pm3-like* genes [7,101]. Here, the alleles *Pm3j*, *Pm3h*, and *Pm3i* were identical to *Pm3b*, *Pm3d*, and *Pm3c*, respectively. These results enrich our understanding of the resistance mechanism of NLR gene clusters induced by gene replication in wheat. Moreover, NLR clusters could lead to improved pathogen recognition via homo- and heterodimerization/oligomerization of the NLRs encoded by the same cluster [71].

Most NLRs occur as singletons. A study showed that the *Sr35* gene encodes an NLR receptor that, after recognizing *AVRSr35*, an *Avr* protein, conferred near-immunity to *Ug99*, the causal agent of wheat blast and stem rust [102,103]. This report supports that interaction between an effector and NLR confers immunity, based on the *Avr*–R gene-for-gene interaction model, and helps rapidly control pandemics in bread wheat [104]. One study using a cryo-electron microscopic structure demonstrated the complex interaction between *Sr35* and the *AVRSr35* effector of the stem rust pathogen of wheat [105]. The effector directly binds to *Sr35*′s LRRs and forms a pentameric complex called the *Sr35* resistosome. These novel insights into the complex structural interaction helped us generate novel variants of NLRs of barley and wheat that identify *AVRSr35* [105].

## 7. Future Perspectives

Over the years, our knowledge about the molecular mechanisms governing the function of NLR genes has improved. This progress has been largely driven by the advancement of molecular biology techniques and the availability of high-quality genome sequences for both plants and pathogens. The elucidation of these molecular mechanisms will be critical for the rational design and deployment of novel R genes that can impart long-lasting and broad-spectrum resistance against diverse pathogens [106]. One of the key challenges in engineering and deploying novel R genes is to identify the most effective R gene combinations that can provide durable resistance against various pathogens. For this, we need to have a complete idea of the molecular basis of R gene function and the co-evolutionary dynamics between pathogens and host plants [90]. Furthermore, the development of R gene-based resistance strategies should be complemented by implementing integrated pest management approaches that can minimize the selection pressure on pathogens and reduce the likelihood of resistance breakdown. Understanding the molecular underpinnings of R gene function is critical for modifying and deploying novel R genes that can offer long-lasting and broad-spectrum resistance against various plant pathogens [107]. As our knowledge of these mechanisms continues to grow, they hold great promise for the development of innovative strategies to enhance crop protection and improve global food security.

In bread wheat, speciation and domestication occurred remarkably quickly compared to other crops, as reported in [108]. Bread wheat evolved due to the effect of duplications in genes, particularly via WGD, during both speciation and domestication processes. Despite the diversity bottleneck that newly formed allopolyploids experienced, bread wheat differs significantly from its diploid progenitors, which formed much earlier than bread wheat [109]. Compared with the diploid progenitors, bread wheat exhibits broad morphological variation, occupies more diverse ecological habitats, and is spread over a larger area [110]. Recent studies have delivered new insights into the occurrence and consequences of genomic changes induced by gene duplication, particularly whole genome duplication. Despite these advances, there remain many opportunities for novel discoveries in gene duplication and wheat disease resistance research. Addressing the following challenges will be crucial to advancing this field: (1) identifying novel R genes; (2) understanding the role and mechanism via which abiotic and/or biotic factors regulate and manipulate these R genes; (3) comprehending the various mechanisms of gene replication; (4) understanding how gene duplication affects NLR cluster size and NLR number; (5) understanding how gene duplication regulates the abundance and sequence variability of NLRs; and (6) exploring the relationship between gene duplication and potentially neo-functionalized genes.

## Data Availability

No new data were created or analyzed in this study. Data sharing is not applicable to this article.
